# Factors associated with regional differences in healthcare quality for patients with acute myocardial infarction in Japan

**DOI:** 10.1371/journal.pone.0319179

**Published:** 2025-04-16

**Authors:** Shusuke Watanabe, Etsu Goto, Susumu Kunisawa, Yuichi Imanaka

**Affiliations:** 1 Department of Healthcare Economics and Quality Management, Graduate School of Medicine, Kyoto University, Kyoto, Japan,; 2 Depertment of Health Security System, Center for Health Security, Graduate School of Medicine, Kyoto University; London School of Hygiene & Tropical Medicine Centre of Global Change and Health: London School of Hygiene & Tropical Medicine, UNITED KINGDOM OF GREAT BRITAIN AND NORTHERN IRELAND

## Abstract

**Background:**

The quality of healthcare at the regional level is of great interest. Regional differences in the quality of healthcare for cardiovascular diseases have been pointed out in previous research, but the regional factors that influence the quality remain unclear.

**Methods:**

Using the national Diagnosis Procedure Combination (DPC) database, risk-adjusted mortality (RAM) in each secondary medical area (SMA) was derived as an indicator for the quality of the healthcare for patients with acute myocardial infarction (AMI). Factors associated with RAM were analysed using the partial least squares (PLS) regression model.

**Results:**

There is a wide variation in RAM in the SMAs; the maximum value was 0.593 and the minimum value was 1.445. The PLS regression identified two components positively correlated with RAM. The first component (Component 1) positively correlates with the proportion of the elderly within the population and negatively correlates with the number of medical facilities per area, the population density, and the intra-SMA access to centres with a high volume of emergency percutaneous coronary interventions (ePCI). The second component (Component 2) positively correlates with the number of medical facilities per area and negatively correlates with the number of doctors per person and the intra-SMA access to centres with a high volume of ePCI.

**Conclusions:**

There was wide regional variation in the quality of in-hospital AMI treatments. Results suggested the degree of rurality, the sufficiency of medical resources, the access to high-volume ePCI centres, and coordination of healthcare delivery were associated with healthcare quality.

## Introduction

Japanese healthcare is essentially financed by social health insurance (SHI) but is mainly provided by private bodies [1]. To improve coordination between medical facilities owned by various bodies, prefectural governments are responsible for establishing regional medical plans [2]. Such plans set goals and action plans to improve regional health systems, and each medical facility should make efforts to cooperate with these plans [2].

Reforms to promote regional medical coordination have become more important in recent years [3] due to the rapid changes in population structure [4]. An aging population presents changes in medical needs and a decrease in the working population, resulting in the constraint of medical resources [5,6]. Consequently, reform intends to accelerate regional coordination to improve efficiency.

Along with improving efficiency, quality assurance is a key goal of the regional medical plans [2]. Healthcare quality is defined as “the degree to which health services for individuals and populations increase the likelihood of desired health outcomes and are consistent with current professional knowledge” [7], and this definition can be applied to a regional level. In terms of the assessment of healthcare quality, among many other indicators, risk-adjusted mortality (RAM) has been supposed to reflect quality of care in hospitals and widely used to examine healthcare-quality [8–13].

While regional differences in healthcare quality for acute diseases are of great interest [14–18], little is known about the factors associated with the quality at a regional level. Regarding acute cardiovascular diseases, the presence of more cardiologists [19] and a lower time from the onset of disease to arrival at the hospital [20] were suggested to have a positive association with outcomes at the hospital level. However, from the viewpoint of structure of healthcare systems, the evidence suggests the trade-off between access and volume at the regional level. An area where high volume centres provide large proportion of medical treatments might spoil access to the treatments because of small number of facilities. Hence, share of high-volume centres should be of great interest at the regional level. Moreover, the outcomes of cardiovascular diseases have also been suggested to have regional differences, associated with social and healthcare factors [21,22]. Behavioural risks and medical spending are reported to have a positive impact on outcomes [21,22], though as these studies directly focused on all of the residents’ outcomes, it is uncertain whether the suggested factors are associated with healthcare quality or the residents’ health conditions before arriving at the hospital.

As mentioned in the previous sections, many medical and social factors, including the residents’ features, are associated with the quality of healthcare for cardiovascular diseases. However, the relationship between the regional factors and the structure to influence the quality are ambiguous. These many regional factors are intercorrelated. This study aims to clarify the regional differences in healthcare quality for acute myocardial infarctions (AMIs) and to disentangle the regional factors associated with healthcare quality.

## Methods

### Secondary medical areas in Japan

In the regional medical plans, secondary medical areas (SMAs) are designated units that provide general in-hospital healthcare [2,23]. As of January 2022, there are 335 secondary medical areas. In Japan, the 52 area units for the management of more advanced treatments are designated as tertiary medical areas, while more primary treatments are managed within smaller areas, across approximately 1,700 districts [23]. We selected secondary medical areas as units of analysis given they are the basic units in the coordination of in-hospital care. For consistency of analysis, we utilised the area setting as of January 2022.

### Data sources

We utilised data from three databases, adding to publicly open data. The first two databases consisted of Diagnosis Procedure Combination (DPC) data, one of which is managed by the Ministry of Health, Labour, and Welfare (MHLW), hereinafter referred to as the MHLW DPC database. DPC data are used for the Diagnosis Procedure Combination/Per-Diem Payment System (DPC/PDPS)—a reimbursement system set up for acute care—and are also used to improve systems and policies in Japan [24]. Hence, the data include patient clinical information such as disease severity, adding to the information of clinical procedures [24]. From this MHLW DPC database, aggregated data can be obtained for research purposes.

The second database utilised was that of the nationwide database of DPC data submitted from participating hospitals for research, hereinafter referred to as the nationwide DPC research database. While not all hospitals included in the MHLW DPC database participated in the nationwide DPC research database, the individual data are available for research.

The third database is the National Database of Health Insurance Claims and Specific Health Check-ups of Japan (NDB), which is a national database of SHI claims data and includes information on all the medical procedures covered by the SHI in Japan [25,26].

### Regional factors associated with the quality of AMI healthcare

The factors associated with differences in the indicator values of healthcare quality for AMI patients across regions were analysed using the partial least squares (PLS) regression model with regional variables.

#### Regional quality indicators of healthcare for AMI patients.

As a quality indicator of care for patients with AMIs, RAM was derived as below. First, based on the data from the nationwide DPC research database, a prediction model for in-hospital mortality of patients admitted with diagnoses of AMI was produced. AMI was defined as I21.x－I24.x using the International Statistical Classification of Diseases and Related Health Problems. The employed predictors at admission were sex, age, body mass index (BMI), Killip classification, and the Japan Coma Scale [27,28]. Data of patients admitted between July 2010 and March 2018 with diagnoses of AMIs were included for the establishment of the model. A multilevel logistic regression model with random intercepts of hospital identifiers was employed to account for clustering within the facilities [29]. Second, aggregated data of case volume, expected deaths, and observed deaths of in-hospital AMI patients in each SMA from fiscal year (FY) 2016 to FY 2018 (from April 2016 to March 2019) were obtained from the MHLW DPC database. The expected deaths were calculated by the derived prediction model. Third, the ratio of expected deaths to observed deaths in each area from FY 2016 to FY 2018 was calculated as RAM, a regional quality indicator of the AMI treatment, with risk adjustment through indirect standardization.

#### Regional variables.

The variables of regional medical resources, resident features, and basic geographical features are included as explanatory variables, because various factors are of interest to influence the quality at the regional level [14–17]. The details of employed variables are presented in S1 Table. All the variables were gathered from publicly available data, excluding the indicator of intra-SMA share of centres with a high volume of emergency percutaneous coronary interventions (ePCI), hereinafter, referred to as the Share of High-volume Centres. This indicator is introduced from the viewpoint of the regional health systems as stated in the previous section. This indicator was derived using data extracted from the NDB, which includes all the medical procedures covered by the SHI. The ePCIs were defined as K546-1, K546-2, K549-1, and K549-2 of the claim codes. The median case number of emergency PCIs of hospitals where at least one ePCI occurred from FY 2016 to FY 2018 was set as the threshold. Then, the hospitals with case numbers over the threshold were defined as the high-volume ePCI centres, and an ePCI share of high-volume ePCI centres in each SMA was calculated as the indicator.

#### Imputation of missing death numbers in SMAs.

For privacy reasons, when the observed deaths were smaller or equal to nine, the exact numbers were masked in the available data, resulting in missing RAM values. These missing values were non-random, meaning usual methods is not applicable. We performed imputation as follows, utilising association between the crude mortality and the case volumes [30]. First, the association between AMI case volumes and crude mortality was analysed. Then, based on the association, the crude mortality for SMAs with missing values was estimated and imputed. Specifically, the weighted mean of the observed crude mortality in SMAs was used for the estimation, where case volumes were under the 90^th^ percentile of the distribution of case volume of SMAs with missing values. Missing observed deaths were calculated using the imputed crude mortality, and when the estimated number of deaths was larger than the possible maximum value of nine, nine was imputed. Finally, RAM was derived from the imputed observed deaths.

#### Regression models.

To analyse the association between regional factors and the quality of healthcare for AMI patients, regression analyses with the dependent variable of RAM were performed. Since the regional factors of interest in this study were intended to be interdependent, the PLS regression was undertaken as the main analysis. PLS regression was employed for epidemiological data with multicollinearity [31–35]. PLS regression can provide more stable results than classical ordinary least squares regression especially when sample size is small [36]. Ordinary least squares (OLS) regression was performed to examine multicollinearity.

#### Statistical analyses.

The number of components in the PLS regression was set by evaluating the cumulative explained variance of the dependent variable by components. To avoid explanatory variables with large absolute values dominating the analysis, data centring and scaling were performed before PLS regression [31].

SAS software version 9.4 (SAS Institute Inc., Cary, NC) was used for setting up the prediction model for AMI patient mortality. PLS regression, other statistical analyses, and plotting were carried out using R version 4.0.5 (R Foundation for Statistical Computing, Vienna, Austria) with the R package “*pls*” version 2.8–0 [37].

#### Sensitivity analyses.

In the PLS regression, sensitivity analyses were undertaken for the imputation of missing values of observed deaths and the Share of high-volume centres. For the imputation of missing values of observed deaths, in the first sensitivity analysis, the crude mortality used for estimation was the weighted mean of the mortality in SMAs, where case volumes were under the maximum value, rather than the 90^th^ percentile, of the case volumes of SMAs with missing values. The second sensitivity analysis was undertaken, limited to the SMAs where exact observed deaths were obtained.

For the Share of High-volume Centres, in the first sensitivity analysis, SMAs where no ePCIs were performed were removed. In these areas, access was not considered to be high but the coordination between hospitals was hardly considered as completed in the SMAs. In the second sensitivity analysis, the indicator was set to one if there was only one hospital that undertook ePCIs in a SMA, even when case volumes of those hospitals were under the threshold. These hospitals are considered to have a central role in the SMAs.

#### Ethical statement.

This study was conducted in accordance with the principles of the Declaration of Helsinki and the study was approved by the Ethics Committee, Kyoto University Graduate School and Faculty of Medicine (approval numbers: R0135, R1389, and R2062) with a waiver of informed consent prior to data collection.

## Results

### Quality indicators of AMI treatment and the characteristics of medical areas

For the establishment of the prediction model, 394,087 patients were included and there were 55,727 in-hospital deaths between July 2010 and March 2018. The c-statistic of the derived prediction model was 0.9177. The observed and predicted deaths for each SMA were made public on the website of the Department of Healthcare Economics and Quality Management, Graduate School of Medicine, Kyoto University (http://med-econ.umin.ac.jp/fH9VdGtT/).

**Fig 1:**
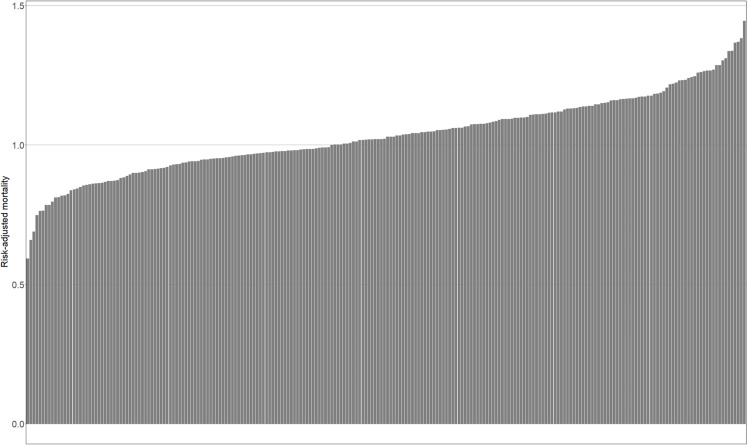
Risk-Adjusted Mortality (RAM) of Secondary Medical Areas (SMAs). The RAM of SMAs is plotted in ascending order.

Table 1 shows the characteristics of the SMAs. The median area and population of the SMAs were 855.66 km^2^ and 224,369, respectively. There were missing RAM values for 103 SMAs and in 32 SMAs there were no facilities undertaking ePCIs. Fig 1 shows the sorted bar plot of the SMAs’ RAM. There is wide variation in RAM; the maximum value was 0.593 and the minimum value was 1.445. The ratio of the maximum to the minimum was 2.436.

In the NDB, 185,900 emergency PCIs were performed in 1,443 hospitals from FY 2016 to FY 2018. The median case number of emergency PCIs of hospitals where at least one ePCI occurred was 89, which was set as the threshold for calculation of the Share of High-volume Centres. In 76 SMAs, the total number of ePCI was less than 89.

**Table 1:** Characteristics of SMAs.

	n = 335
Medical Resource	
the Share of High-volume Centres (median [IQR])	0.86 [0.48, 0.96]
Number of all physicians per resident (/100,000 persons) (median [IQR])	181.07 [150.96, 225.35]
Number of cardiologists per resident (/100,000 persons) (median [IQR])	7.33 [5.16, 10.29]
Number of cardiovascular surgeons per resident (/100,000 persons) (median [IQR])	1.51 [0.00, 2.62]
Number of beds per resident (/100,000 persons) (median [IQR])	703.16 [574.27, 866.38]
Number of emergency hospitals per area (/km2) (median [IQR])	0.01 [0.00, 0.02]
Number of hospitals per area (/km2) (median [IQR])	0.02 [0.01, 0.04]
Number of clinics per area (/km2) (median [IQR])	0.19 [0.07, 0.53]
Medical expenditure per person (1,000 yens) (median [IQR])	579.61 [530.69, 645.93]
Residents’ features	
Population proportion, under 14 y/o (median [IQR])	0.12 [0.11, 0.13]
Population proportion, 65–74 y/o (median [IQR])	0.15 [0.14, 0.16]
Population proportion, over 75 y/o (median [IQR])	0.15 [0.13, 0.18]
Proportion of people working (median [IQR])	0.48 [0.46, 0.50]
Proportion of people working in the first industry (median [IQR])	0.03 [0.01, 0.06]
Proportion of people working in the second industry (median [IQR])	0.11 [0.09, 0.14]
Proportion of people working in the third industry (median [IQR])	0.30 [0.29, 0.32]
Taxable income per person (1,000 yens) (median [IQR])	1191.23 [1013.90, 1357.37]
Basic features	
Population (100,000 persons) (median [IQR])	2.24 [1.04, 4.77]
Area (km2) (median [IQR])	855.66 [434.46, 1405.56]
Proportion of habitable area (median [IQR])	0.37 [0.25, 0.56]
Population density (/ha) (median [IQR])	2.46 [0.94, 6.90]
Quality of AMI healthcare	
RAM (median [IQR])	1.03 [0.95, 1.13]

SMA, secondary medical area; IQR, Interquartile range; ePCI, emergency percutaneous coronary intervention; y/o, years old; AMI, acute myocardial infarction; RAM, risk adjusted mortality.

### SMAs’ AMI Case Volumes and Crude Mortality

S1 Fig plots the SMAs’ AMI case volumes and crude mortality. There is a strong negative correlation between case volumes and crude mortality (correlation coefficient: -0.412; 95% confidence interval: -0.467 to -0.353), justifying the imputation of crude mortality based on the distribution of case volume. Missing Death numbers were observed in 103 SMAs and the imputation was performed in these areas.

### Regression analyses

OLS regression gave large Variance Inflation Factors (VIFs) for some variables. Specifically, 50.7 for the proportion working in the second industry, 48.8 for the number of hospitals per area, and 39.5 for the proportion working in the first industry. These large VIFs demonstrate strong multicollinearity in the data.

With the PLS regression, the cumulative explained variances of the dependent variable by components 1–5 were 0.1765, 0.2137, 0.2223, 0.2246, and 0.2269. More than two components could create a minor difference to the explained variance, and therefore, we chose two as the number of components.

**Fig 2:**
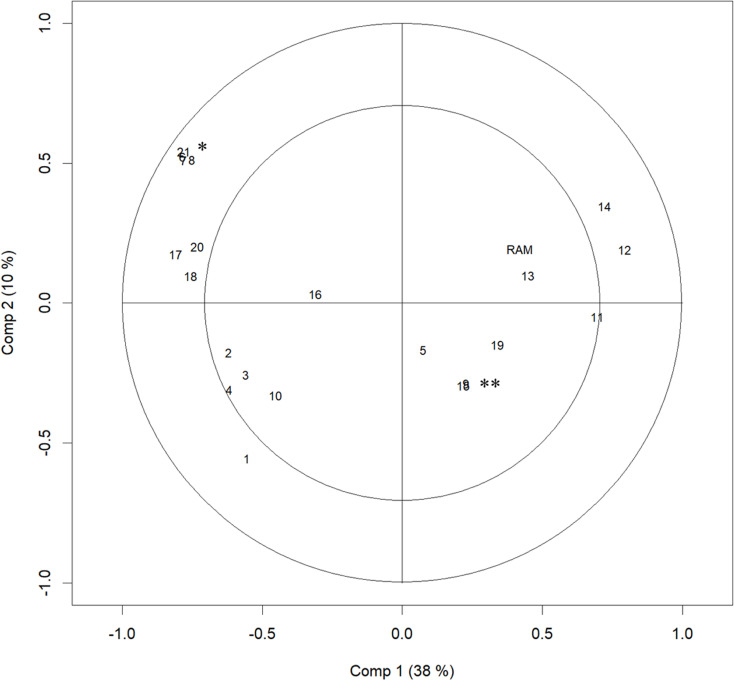
Correlation Loading Plot of PLS Regression Results. The plot of numbers indicates the loadings of variables to Component 1 and 2. The plot of RAM indicates its correlation with Components 1 and 2. The distance from the origin indicates the variance of the variable explained by Components 1 and 2. The inner and the outer circles indicate 50% and 100% explained variance, respectively. Each number indicates as following aspects: 1, the Share of High-volume Centres; 2, number of all physicians per resident; 3, number of cardiologists per resident; 4, number of cardiovascular surgeons per resident; 5, number of beds per resident; 6, number of emergency hospitals per area; 7, number of hospitals per area; 8, number of clinics per area; 9, medical expenditure per person; 10, population proportion, under 14 y/o; 11, population proportion, 65–74 y/o; 12, population proportion, over 75 y/o; 13, proportion of people working; 14, proportion of people working in the first industry; 15, proportion of people working in the second industry; 16, proportion of people working in the third industry; 17, taxable income per person; 18, population; 19, area; 20, proportion of habitable area; 21, population density. * indicates numbers of 6, 7, 8, and 21. ** indicates numbers of 9 and 15. PLS, partial least squares; ePCI, emergency percutaneous coronary intervention; y/o, years old; RAM, risk-adjusted mortality.

Fig 2, Table 2, and S2 and S3 Fig show the results of the PLS regression. S2 and S3 Fig show the plot of the SMAs’ RAM with the scores of Component 1 and with those of component 2. There were positive correlations between RAM and the scores of Components 1 and 2. Since low RAM indicates good quality of AMI care, small scores of Components 1 and 2 were supposed to correlate with good quality.

**Table 2:** Loadings and coefficient of the regional variables from the PLS regression analysis.

	Loadings		Coefficient
	Component 1	Component 2	
Medical Resource			
the Share of High-volume Centres	-0.203	-0.404	-0.0764500
Number of all physicians per resident (/100,000 persons)	-0.226	-0.128	-0.0001611
Number of cardiologists per resident (/100,000 persons)	-0.204	-0.186	-0.0022189
Number of cardiovascular surgeons per resident (/100,000 persons)	-0.226	-0.225	-0.0103527
Number of beds per resident (/100,000 persons)	0.027	-0.122	0.0000145
Number of emergency hospitals per area (/km2)	-0.286	0.381	0.0119200
Number of hospitals per area (/km2)	-0.285	0.371	0.0012809
Number of clinics per area (/km2)	-0.274	0.372	0.0013376
Medical expenditure per person (1,000 yens)	0.083	-0.208	-0.0000796
Residents’ features			
Population proportion, under 14 y/o	-0.165	-0.239	-0.8500749
Population proportion, 65–74 y/o	0.254	-0.035	0.3062721
Population proportion, over 75 y/o	0.290	0.139	0.5046442
Proportion of people working	0.164	0.071	0.6195301
Proportion of people working in the first industry	0.263	0.252	0.7702309
Proportion of people working in the second industry	0.079	-0.214	-0.1200035
Proportion of people working in the third industry	-0.114	0.024	0.0590919
Taxable income per person (1,000 yens)	-0.296	0.128	-0.0000205
Basic features			
Population (100,000 persons)	-0.276	0.070	-0.0022859
Area (km2)	0.124	-0.108	-0.0000013
Proportion of habitable area	-0.267	0.147	-0.0297865
Population density (/ha)	-0.285	0.394	0.0000179

PLS, partial least squares; ePCI, emergency percutaneous coronary intervention; y/o, years old.

Fig 2 and Table 2 show each variable’s coefficient and loadings with Components 1 and 2. With Component 1, the population proportion of the elderly (65–74 y/o and 75+ y/o), and the proportion working in the first industry had a positive loading, and the number of medical facilities (emergency hospitals, hospitals, and clinics) per area, the population density, the taxable income per persons, the population, and the proportion of habitable area had a negative loading. With Component 2, the numbers of medical facilities (emergency hospitals, hospitals, and clinics) per area and the population density had a positive loading, while the Share of High-volume Centres and the number of physicians (i.e., the number of all physicians, cardiologists, and cardiovascular surgeons) per person had a negative loading.

**Fig 3:**
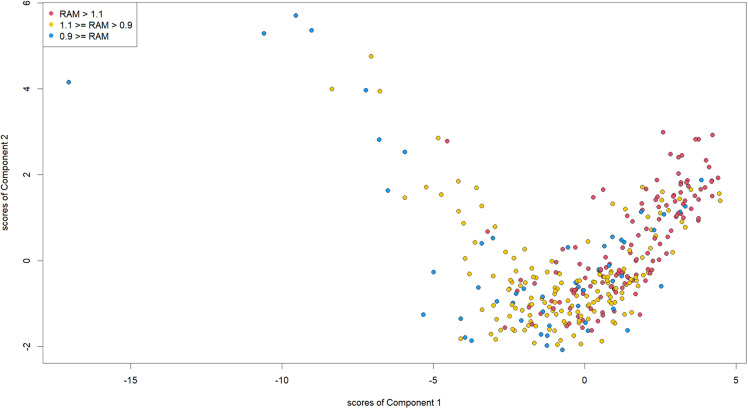
SMA Scores for Components 1 and 2. Points indicate SMAs’ scores of Components 1 and 2. Red points indicate SMAs where RAM is over 1.1. Green points indicate SMAs where RAM is 0.9–1.1. Blue points indicate SMAs where RAM is equal to or under 0.9. PLS, partial least squares; RAM, risk-adjusted mortality; SMA, secondary medical area.

Fig 3 plots SMAs’ scores of Components 1 and 2. SMAs in which the scores of Component 1 were close to the smaller and larger ends show relatively large scores for Component 2.

S2–S5 Tables show the loadings and the coefficients resulting from sensitivity analysis. There was no substantial difference with the main analysis.

## Discussion

While we adjust regional variation of risk of patients at admission, there was substantial regional variation in the mortality of AMI patients. There was a 2.436 times difference in the risk-adjusted in-hospital mortality. Through PLS regression analysis, we identified two components that influence the regional quality of healthcare for AMI patients, as follows.

Component 1 can be interpreted as the degree of rurality, given the features of having a large elderly proportion [38], low population density, low income [39], and poor medical human resources [40–42] match the characteristics of rural areas in Japan. The suggested negative influence of rurality on healthcare quality is consistent with previous findings [17]. In addition, having sufficient medical resources was suggested to improve healthcare quality for AMI patients at a hospital level [19], which was consistent with our regional-level investigation. The proportion of the elderly, whose loading for Component 1 was positive, can be interpreted as one aspect of the insufficiency of medical resources. Large proportions of the elderly mean a large demand for healthcare, resulting in more burden on the system and a scarcity of medical resources.

Component 2 positively correlated with the population density and the number of medical facilities per area, but negatively correlated with the number of physicians per person and the Share of High-volume Centres, which cannot be explained by the degree of rurality. One possible explanation of Component 2 is that it reflects not only the degree of richness of medical resources but also the integration of healthcare delivery–coordinated healthcare delivery. Less share of high-volume ePCI centres and more medical facilities per area possibly mean that healthcare provision is fragmented and less integrated, meaning healthcare delivery is not well-coordinated. Although the observational evidence at a regional level was scarce, fragmentation of the healthcare system has been theoretically suggested to reduce performance [43].

Loadings of the variables can explain the score plot of the SMAs. When the population density and number of facilities per area are large, the score of Component 1 tends to be small, but the score of Component 2 tends to be large. In fact, SMAs in which the scores of Component 1 were close to the lower end show relatively high scores in Component 2. In contrast, when the two indicators of the Share of High-volume Centres and physicians per person are low, the scores of Component 1 and 2 tend to be high. Indeed, SMAs in which the scores of Component 2 were close to the higher end show relatively high scores for Component 2.

Policy actions are needed to address the unwarranted disparity in healthcare quality; the discussion above suggests that the approaches required to improve quality are different between rural and urban areas. In rural areas, the required policy actions should target the reduction of Components 1 and 2 simultaneously; the score plot shows the positive correlation between Components 1 and 2 for SMAs with high scores for Component 1. For example, medical resources are considered to be sensitive to health policies and increasing medical resources can reduce the scores in both Components 1 and 2. Uneven distribution of medical professionals is recognised as an important issue in Japanese health policy and our results add further rationale to solving regional disparities. At the same time, increasing the Share of High-volume Centres can also decrease the scores of Components 1 and 2.

In contrast, in urban areas, the importance of utilizing medical resources—a part of Component 2—should be stressed more. From the perspective of addressing the uneven distribution of medical resources, policies to decrease the scores of Component 1 in urban areas is difficult to justify. However, there is the possibility of increasing efficiency, including increasing the Share of High-volume Centres; the score plot shows that a high percentage of SMAs with low scores for Component 1 had high scores for Component 2. However, it should be noted that compared with Component 1, the explained variance in RAM by Component 2 was small, meaning the effect of reducing fragmentation is less significant than decreasing rurality.

The loadings of the variables, shown in Fig 2, highlight the importance of the share of high-volume ePCI centres. That indicator had negative loadings both for Components 1 and 2, and the distance from the origin was large, meaning there was a large correlation with Components 1 and 2. This result suggests the potential of increasing share of high-volume ePCI centres, to improve healthcare quality. Centralization of ePCIs may have potential to improve quality of care for AMIs. In fact, the hub-and-spoke model—where a network between centres providing resource-intensive services (i.e., the hub) and facilities providing more primary services (i.e., the spokes) is established—has been posited as an effective way to improve outcomes [44,45], which is consistent with our results.

Policy implications from the results can be summarized as below. Firstly, at the national level, uneven distribution of medical resources should be stressed. In fact, the degree of rurality, including resource richness, was associated with quality of healthcare for AMI. Secondly, at the intra-SMA level, promoting coordination of healthcare, including centralization of ePCIs, should be emphasized.

This study has several limitations. First, although the DPC data for the majority of patients who received acute case treatment was included in the MHLW DPC database [24], the calculation of each SMA’s RAM for the patients whose DPC data were available could introduce selection bias. Second, as described, there were missing RAM values due to data availability. We performed imputation as justified by the data, and sensitivity analyses showed that the conclusion of the study was valid, but the potential bias due to the missing values cannot be denied. Third, this study did not consider the many unobservable factors that can influence healthcare quality. For instance, the skill level of health professionals, on which data are not available at the regional level, can improve healthcare quality. In addition, the results implied the fragmentation of healthcare provision might influence the quality, but many factors other than those involved in this study can also be associated with the fragmentation. Forth, some SMAs might not be appropriate as a unit of analysis. Although SMAs were publicly designated area for in-hospital care, in some SMAs, numbers of ePCIs undertaken were small. Lastly, RAM may reflect other factors than health-care-quality in hospitals [46,47]. Further research is warranted to clarify the mechanism that produces good quality of healthcare at a regional level.

## Conclusion

There was wide regional variation in the quality of in-hospital healthcare for AMI patients. Results suggest that, at a regional level, the degree of rurality, the sufficiency of medical resources, access to high-volume ePCI centres, and coordination of healthcare delivery were associated with healthcare quality for AMI patients.

## Supporting information

S1 Fig SMAs’ AMI Case Numbers and Crude Mortality. SMA, secondary medical area; AMI, acute myocardial infarction.
(TIF)

S2 Fig Component 1 Scores and RAM of SMAs. Scores of Component 1 in the horizontal axis and RAM in the vertical axis. SMA, secondary medical area; RAM, risk-adjusted mortality; corr., correlation; coef., coefficient; CI, confidence interval.
(TIF)

S3 Fig Component 2 Scores and RAM of SMAs. Scores of Component 2 in the horizontal axis, and RAM in the vertical axis. SMA, secondary medical area; RAM, risk-adjusted mortality; corr., correlation; coef., coefficient; CI, confidence interval.
(TIF)

S1 Table Reference of regional variables. y/o, years old; MHLW, Ministry of Health, Labour, and Welfare; MIAC, Ministry of Internal Affairs and Communication. * the way of calculation was described in the Methods section.
(DOCX)

S2 Table Loadings and coefficient of the regional variables from the PLS regression analysis of the first sensitivity analysis for the imputation for missing values of observed deaths (the crude mortality proportion used for the estimation was the proportion in SMAs where case volumes were under the maximum value of the case volumes of SMAs with missing values). PLS, partial least squares; SMA, secondary medical area; PLS, partially least squares; ePCI, emergency percutaneous coronary intervention; y/o, years old.
(DOCX)

S3 Table Loadings and coefficient of the regional variables from the PLS regression analysis of the second sensitivity analysis for the imputation for missing values of observed deaths (limiting to the SMAs where exact observed deaths were obtained). PLS, partial least squares; SMA, secondary medical area; PLS, partially least squares; ePCI, emergency percutaneous coronary intervention; y/o, years old.
(DOCX)

S4 Table Loadings and coefficient of the regional variables from the PLS regression analysis of the first sensitivity analysis for the ePCI-large-hospitals share (SMAs where no ePCIs were performed were removed). SMA, secondary medical area; PLS, partially least squares; ePCI, emergency percutaneous coronary intervention; y/o, years old.
(DOCX)

S5 Table Loadings and coefficient of the regional variables from the PLS regression analysis of the second sensitivity analysis for the ePCI-large-hospitals share (the indicator was set to one if there is only one hospital which undertook ePCIs in a SMA even when case volumes of those hospitals were under the threshold). PLS, partial least squares; SMA, secondary medical area; PLS, partially least squares; ePCI, emergency percutaneous coronary intervention; y/o, years old.
(DOCX)
