# Supplementary figures and images for: Factors associated with regional differences in healthcare quality for patients with acute myocardial infarction in Japan
Source: PLoS One. 2025 Apr 16;20(4):e0319179. doi: 10.1371/journal.pone.0319179 (PMC12002444; doi:10.1371/journal.pone.0319179)

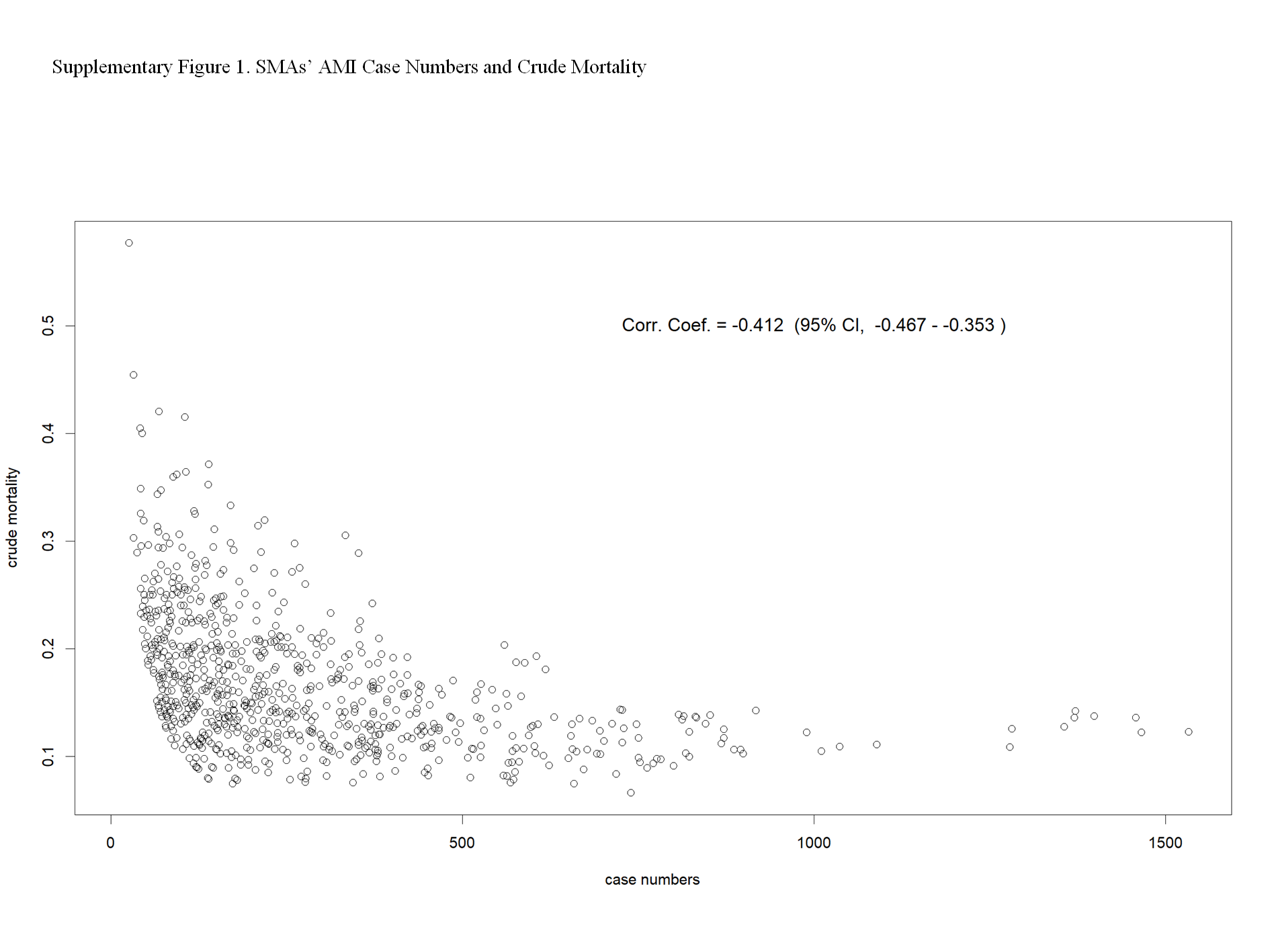

Supplement: S1 Fig — SMA, secondary medical area; AMI, acute myocardial infarction. (TIF) [file pone.0319179.s001.TIF]

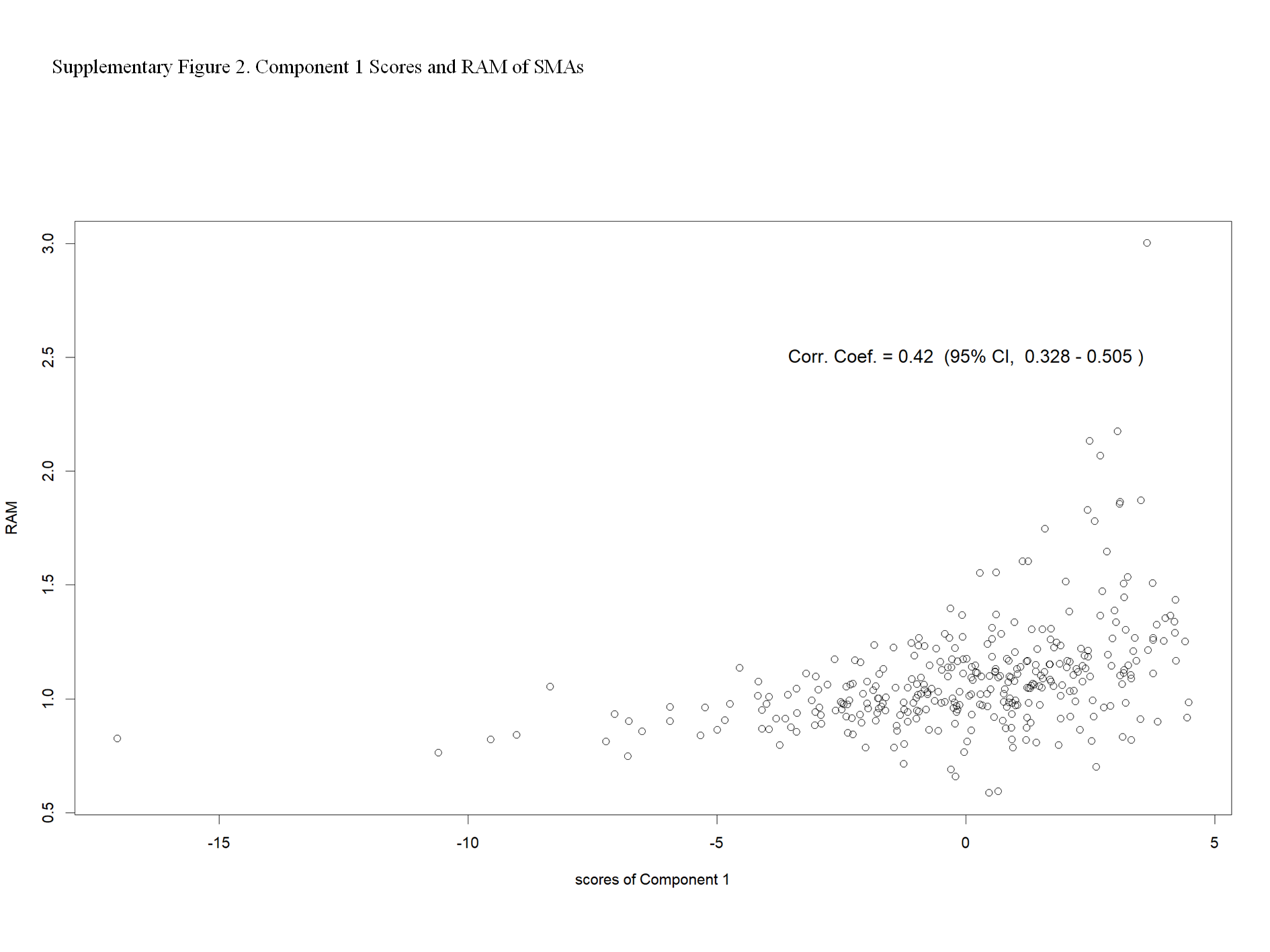

Supplement: S2 Fig — Scores of Component 1 in the horizontal axis and RAM in the vertical axis. SMA, secondary medical area; RAM, risk-adjusted mortality; corr., correlation; coef., coefficient; CI, confidence interval. (TIF) [file pone.0319179.s002.TIF]

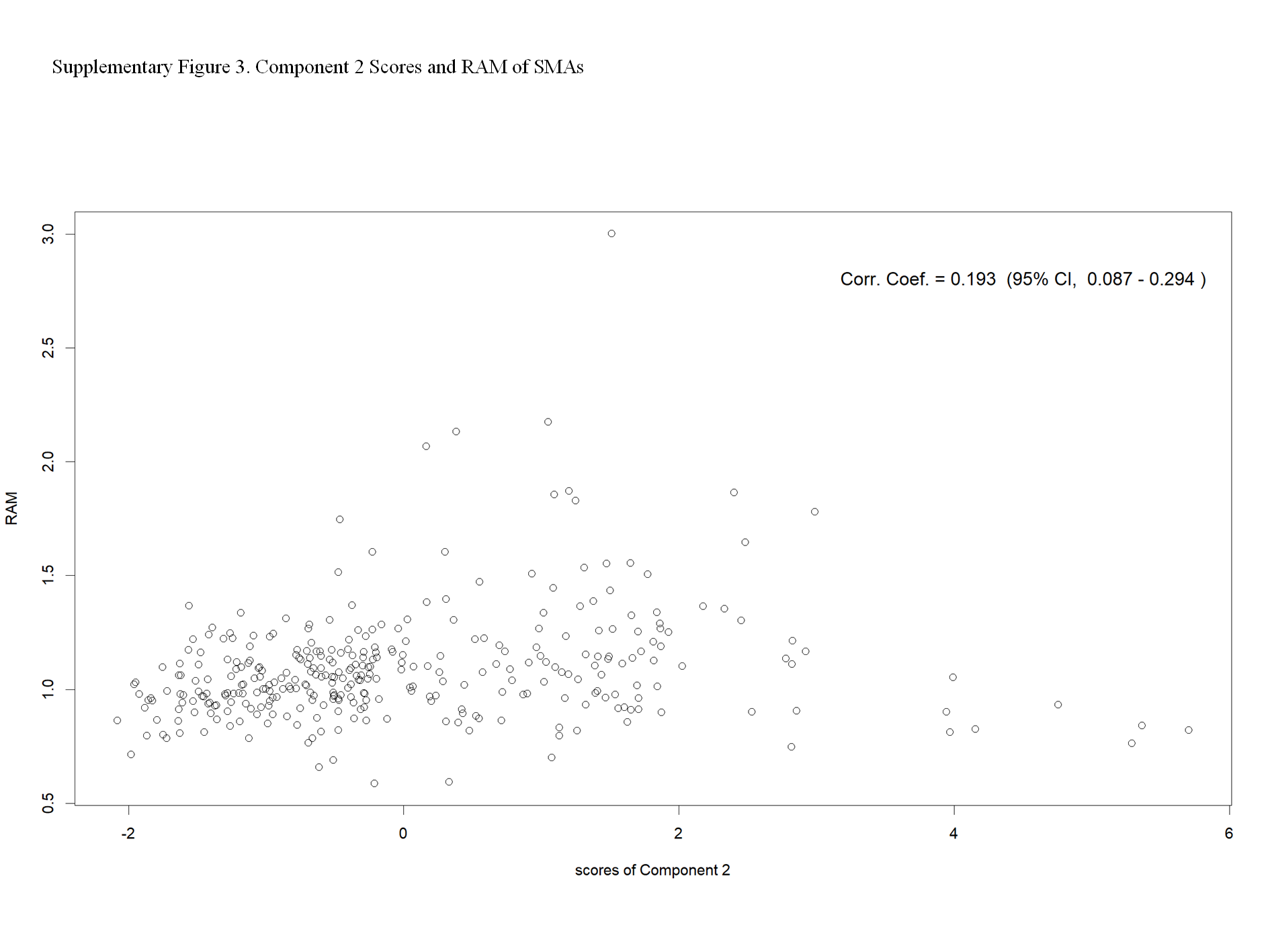

Supplement: S3 Fig — Scores of Component 2 in the horizontal axis, and RAM in the vertical axis. SMA, secondary medical area; RAM, risk-adjusted mortality; corr., correlation; coef., coefficient; CI, confidence interval. (TIF) [file pone.0319179.s003.TIF]
