# Supplementary material for: Factors associated with regional differences in healthcare quality for patients with acute myocardial infarction in Japan
Source: PLoS One. 2025 Apr 16;20(4):e0319179. doi: 10.1371/journal.pone.0319179 (PMC12002444; doi:10.1371/journal.pone.0319179)
Supplement: S1 Table — y/o, years old; MHLW, Ministry of Health, Labour, and Welfare; MIAC, Ministry of Internal Affairs and Communication. * the way of calculation was described in the Methods section. (DOCX) [file pone.0319179.s004.docx]

Supplementary Table 1. Reference of regional variables

|  | Fiscal Year | Reference | |  |
| --- | --- | --- | --- | --- |
| Medical Resource |  |  |  |  |
| the Share of High-volume Centres | 2016-2018 | National Database of Health Insurance Claims and Specific Health Checkups of Japan * |  |  |
| Number of all physicians per resident  (/100,000 persons) | 2016 | Survey of Physicians, Dentists, and Pharmacists (MHLW) |  |  |
| Number of cardiologists per resident  (/100,000 persons) | 2016 | Survey of Physicians, Dentists, and Pharmacists (MHLW) |  |  |
| Number of cardiovascular surgeons per resident  (/100,000 persons) | 2016 | Survey of Physicians, Dentists, and Pharmacists (MHLW) |  |  |
| Number of beds per resident  (/100,000 persons) | 2016 | Survey of Medical Facilities (MHLW) |  |  |
| Number of emergency hospitals per area (/km2) | 2017 | Public data of the reports of the beds functions (MHLW) |  |  |
| Number of hospitals per area (/km2) | 2016 | Survey of Medical Facilities (MHLW) |  |  |
| Number of clinics per area (/km2) | 2016 | Survey of Medical Facilities (MHLW) |  |  |
| Medical expenditure per person (1,000 yens) | 2018 | Regional difference of the medical expenditure (MHLW) |  |  |
| Residents' features |  |  |  |  |
| Population proportion, under 14 y/o | 2017 | Population, demographics, and the number of households  from Basic Resident Register (MIAC) |  |  |
| Population proportion, 65-74 y/o | 2017 | Population, demographics, and the number of households  from Basic Resident Register (MIAC) |  |  |
| Population proportion, over 75 y/o | 2017 | Population, demographics, and the number of households  from Basic Resident Register (MIAC) |  |  |
| Proportion of people working | 2015 | National Census (MIAC) |  |  |
| Proportion of people working in the first industry | 2015 | National census (MIAC) |  |  |
| Proportion of people working in the second industry | 2015 | National census (MIAC) |  |  |
| Proportion of people working in the third industry | 2015 | National census (MIAC) |  |  |
| Taxable income per person (1,000 yens) | 2015 | Survey of taxes of shi, ku, machi, mura (MIAC) |  |  |
| Basic features |  |  |  |  |
| Population (100,000 persons) | 2017 | Population, demographics, and the number of households  from Basic Resident Register (MIAC) |  |  |
| Area (km2) | 2017 | Survey of area of shi, ku, machi, mura (MIAC) |  |  |
| Proportion of habitable area | 2017 | Survey of area of shi, ku, machi, mura (MIAC) |  |  |
| Population density (/ha) | 2017 | Survey of taxes of shi, ku, machi, mura (MIAC), Population, demographics, and the number of households  from Basic Resident Register (MIAC) |  |  |
| Quality of AMI healthcare |  |  |  |  |
| RAM (median [IQR]) | 2016-2018 | -* |  |  |
| y/o, years old; MHLW, Ministry of Health, Labour, and Welfare; MIAC, Ministry of Internal Affairs and Communication * the way of calculation was described in the Methods section. | | | | |
