# Supplementary material for: Factors associated with regional differences in healthcare quality for patients with acute myocardial infarction in Japan
Source: PLoS One. 2025 Apr 16;20(4):e0319179. doi: 10.1371/journal.pone.0319179 (PMC12002444; doi:10.1371/journal.pone.0319179)
Supplement: S2 Table — PLS, partial least squares; SMA, secondary medical area; PLS, partially least squares; ePCI, emergency percutaneous coronary intervention; y/o, years old. (DOCX) [file pone.0319179.s005.docx]

Supplementary Table 2. Loadings and coefficient of the regional variables from the PLS regression analysis of the first sensitivity analysis for the imputation for missing values of observed deaths (the crude mortality proportion used for the estimation was the proportion in SMAs where case volumes were under the maximum value of the case volumes of SMAs with missing values)

|  | Loadings | | Coefficient |
| --- | --- | --- | --- |
|  | Component 1 | Component 2 |  |
| Medical Resource |  |  |  |
| the Share of High-volume Centres | -0.192 | -0.343 | -0.0414727 |
| Number of all physicians per resident (/100,000 persons) | -0.229 | -0.226 | -0.0001314 |
| Number of cardiologists per resident (/100,000 persons) | -0.204 | -0.270 | -0.0018010 |
| Number of cardiovascular surgeons per resident (/100,000 persons) | -0.224 | -0.278 | -0.0076604 |
| Number of beds per resident (/100,000 persons) | 0.019 | -0.242 | -0.0000008 |
| Number of emergency hospitals per area (/km2) | -0.289 | 0.344 | -0.0188681 |
| Number of hospitals per area (/km2) | -0.288 | 0.331 | -0.0156930 |
| Number of clinics per area (/km2) | -0.278 | 0.327 | 0.0003380 |
| Medical expenditure per person (1,000 yens) | 0.077 | -0.317 | -0.0000678 |
| Residents' features |  |  |  |
| Population proportion, under 14 y/o | -0.155 | -0.178 | -0.5643065 |
| Population proportion, 65-74 y/o | 0.246 | -0.095 | 0.1957692 |
| Population proportion, over 75 y/o | 0.278 | 0.068 | 0.3064798 |
| Proportion of people working | 0.169 | 0.191 | 0.5427202 |
| Proportion of people working in the first industry | 0.255 | 0.240 | 0.5596337 |
| Proportion of people working in the second industry | 0.092 | -0.055 | 0.0265096 |
| Proportion of people working in the third industry | -0.117 | -0.059 | -0.0926922 |
| Taxable income per person (1,000 yens) | -0.291 | 0.152 | -0.0000170 |
| Basic features |  |  |  |
| Population (100,000 persons) | -0.273 | 0.064 | -0.0016908 |
| Area (km2) | 0.124 | -0.088 | 0.0000010 |
| Proportion of habitable area | -0.263 | 0.176 | -0.0198652 |
| Population density (/ha) | -0.287 | 0.370 | -0.0000603 |
| PLS, partial least squares; SMA, secondary medical area; PLS, partially least squares; ePCI, emergency percutaneous coronary intervention; y/o, years old. | | | |
|  |  |  |  |
