# Supplementary material for: Factors associated with regional differences in healthcare quality for patients with acute myocardial infarction in Japan
Source: PLoS One. 2025 Apr 16;20(4):e0319179. doi: 10.1371/journal.pone.0319179 (PMC12002444; doi:10.1371/journal.pone.0319179)
Supplement: S4 Table — SMA, secondary medical area; PLS, partially least squares; ePCI, emergency percutaneous coronary intervention; y/o, years old. (DOCX) [file pone.0319179.s007.docx]

Supplementary Table 4. Loadings and coefficient of the regional variables from the PLS regression analysis of the first sensitivity analysis for the ePCI-large-hospitals share (SMAs where no ePCIs were performed were removed)

|  | Loadings | | Coefficient |
| --- | --- | --- | --- |
|  | Component 1 | Component 2 |  |
| Medical Resource |  |  |  |
| the Share of High-volume Centres | -0.160 | -0.376 | -0.0557234 |
| Number of all physicians per resident (/100,000 persons) | -0.221 | -0.206 | -0.0001225 |
| Number of cardiologists per resident (/100,000 persons) | -0.194 | -0.253 | -0.0021187 |
| Number of cardiovascular surgeons per resident (/100,000 persons) | -0.214 | -0.263 | -0.0086165 |
| Number of beds per resident (/100,000 persons) | 0.036 | -0.185 | 0.0000270 |
| Number of emergency hospitals per area (/km2) | -0.304 | 0.317 | -0.0070148 |
| Number of hospitals per area (/km2) | -0.303 | 0.304 | -0.0094333 |
| Number of clinics per area (/km2) | -0.294 | 0.302 | 0.0006723 |
| Medical expenditure per person (1,000 yens) | 0.071 | -0.377 | -0.0001107 |
| Residents' features |  |  |  |
| Population proportion, under 14 y/o | -0.123 | -0.005 | -0.0691534 |
| Population proportion, 65-74 y/o | 0.234 | -0.265 | -0.2726920 |
| Population proportion, over 75 y/o | 0.271 | -0.017 | 0.3106837 |
| Proportion of people working | 0.178 | 0.250 | 0.7138245 |
| Proportion of people working in the first industry | 0.265 | 0.334 | 0.9101322 |
| Proportion of people working in the second industry | 0.106 | -0.045 | -0.0055128 |
| Proportion of people working in the third industry | -0.122 | -0.107 | -0.2078844 |
| Taxable income per person (1,000 yens) | -0.299 | 0.117 | -0.0000274 |
| Basic features |  |  |  |
| Population (100,000 persons) | -0.277 | 0.037 | -0.0018993 |
| Area (km2) | 0.142 | -0.045 | 0.0000015 |
| Proportion of habitable area | -0.266 | 0.209 | -0.0127545 |
| Population density (/ha) | -0.303 | 0.340 | -0.0000343 |
| SMA, secondary medical area; PLS, partially least squares; ePCI, emergency percutaneous coronary intervention; y/o, years old. | | | |
