# Supplementary material for: Factors associated with regional differences in healthcare quality for patients with acute myocardial infarction in Japan
Source: PLoS One. 2025 Apr 16;20(4):e0319179. doi: 10.1371/journal.pone.0319179 (PMC12002444; doi:10.1371/journal.pone.0319179)
Supplement: S5 Table — PLS, partial least squares; SMA, secondary medical area; PLS, partially least squares; ePCI, emergency percutaneous coronary intervention; y/o, years old. (DOCX) [file pone.0319179.s008.docx]

Supplementary Table 5. Loadings and coefficient of the regional variables from the PLS regression analysis of the second sensitivity analysis for the ePCI-large-hospitals share (the indicator was set to one if there is only one hospital which undertook ePCIs in a SMA even when case volumes of those hospitals were under the threshold)

|  | Loadings | | Coefficient |
| --- | --- | --- | --- |
|  | Component 1 | Component 2 |  |
| Medical Resource |  |  |  |
| the Share of High-volume Centres | -0.131 | -0.407 | -0.0972094 |
| Number of all physicians per resident (/100,000 persons) | -0.230 | -0.138 | -0.0001738 |
| Number of cardiologists per resident (/100,000 persons) | -0.206 | -0.202 | -0.0024081 |
| Number of cardiovascular surgeons per resident (/100,000 persons) | -0.226 | -0.232 | -0.0113941 |
| Number of beds per resident (/100,000 persons) | 0.026 | -0.135 | 0.0000169 |
| Number of emergency hospitals per area (/km2) | -0.294 | 0.379 | 0.0212653 |
| Number of hospitals per area (/km2) | -0.292 | 0.370 | 0.0059918 |
| Number of clinics per area (/km2) | -0.282 | 0.368 | 0.0016557 |
| Medical expenditure per person (1,000 yens) | 0.085 | -0.201 | -0.0000870 |
| Residents' features |  |  |  |
| Population proportion, under 14 y/o | -0.166 | -0.258 | -0.9340170 |
| Population proportion, 65-74 y/o | 0.258 | -0.019 | 0.3258465 |
| Population proportion, over 75 y/o | 0.292 | 0.159 | 0.5529598 |
| Proportion of people working | 0.167 | 0.077 | 0.6598186 |
| Proportion of people working in the first industry | 0.261 | 0.243 | 0.8615148 |
| Proportion of people working in the second industry | 0.086 | -0.204 | -0.1541198 |
| Proportion of people working in the third industry | -0.115 | 0.029 | 0.0574371 |
| Taxable income per person (1,000 yens) | -0.300 | 0.122 | -0.0000222 |
| Basic features |  |  |  |
| Population (100,000 persons) | -0.279 | 0.068 | -0.0024803 |
| Area (km2) | 0.127 | -0.109 | -0.0000016 |
| Proportion of habitable area | -0.272 | 0.141 | -0.0317202 |
| Population density (/ha) | -0.292 | 0.392 | 0.0000389 |
| PLS, partial least squares; SMA, secondary medical area; PLS, partially least squares; ePCI, emergency percutaneous coronary intervention; y/o, years old. | | | |
